# Pharmacists’ Perspectives on Functional Medication-Use Challenges and Potential Adaptations for People with Disabilities in Saudi Arabia: A Qualitative Study

**DOI:** 10.3390/healthcare14182950

**Published:** 2026-09-10

**Authors:** Fahad S. Alshehri, Nasser M. Alorfi, Nouf M. Alourfi, Wajid Syed

**Affiliations:** 1 Pharmacology and Toxicology Department, College of Pharmacy, Umm Al-Qura University, Makkah 24382, Saudi Arabia; nmorfi@uqu.edu.sa; 2 King Salman Center for Disability Research, Riyadh 11614, Saudi Arabia; 3 Department of Chemistry, Arts and Sciences College, Rabigh Campus, King Abdulaziz University, Jeddah 21589, Rabigh, Saudi Arabia; nalourfi1@kau.edu.sa; 4 Department of Clinical Pharmacy, College of Pharmacy, King Saud University, Riyadh 11451, Saudi Arabia; wali@ksu.edu.sa

**Keywords:** medication safety, disability, pharmacists, pharmaceutical care, medication management

## Abstract

**Background:** People with disabilities may face medication-safety challenges when functional limitations interfere with medication handling, administration, communication, self-management, or continuity of treatment. This study examined pharmacists’ experiences and perceptions of safe medication use among people with disabilities in Saudi Arabia and identified adaptations that may support safer, more independent medication management. **Methods:** A qualitative descriptive study was conducted using a self-administered electronic survey that included structured items on participant characteristics and 15 open-ended questions. Eighteen pharmacists from different practice settings participated, and 17 provided substantive qualitative responses for analysis. Data were analyzed using a codebook thematic analysis approach within a qualitative descriptive orientation, informed by Braun and Clarke’s phases of thematic analysis. Two researchers initially coded the responses independently and subsequently compared and refined the coding framework through discussion. **Results:** Four major themes were identified: (1) functional limitations shape perceived medication-use challenges; (2) pharmacists perceive safety concerns at multiple points in medication use; (3) proposed function-adapted strategies for medication management; and (4) organizational conditions perceived to support function-adapted care. Participants reported challenges with medication packaging, dosage forms, administration devices, medication information, self-management, caregiver dependence, refills, and treatment continuity. They also proposed strategies, including functional assessment, personalized counseling, caregiver education, adapted packaging and dosage forms, medication review, assistive tools, alternative access services, and standardized institutional procedures. **Conclusions:** Pharmacists identified several ways disability-related functional limitations could complicate medication use and proposed potential adaptations at the patient, pharmacist, and organizational levels. These strategies should be viewed as hypotheses and considerations for practice rather than proven medication-safety interventions. Further research that incorporates patient and caregiver perspectives and directly measures medication-use outcomes is needed.

## 1. Introduction

Disability is a major public health and health equity concern that affects individuals’ ability to access, navigate, and benefit from healthcare services [1]. People with disabilities may experience limitations in mobility, dexterity, coordination, or the ability to perform daily activities [2]. These functional limitations can interact with environmental and healthcare system barriers, reducing independence and quality of care. Ensuring equitable healthcare for people with disabilities therefore requires more than physical access to healthcare facilities; services must also be designed to accommodate individual functional needs and to support safe participation in treatment and self-care [1,3]. Medication use is an important but often overlooked aspect of healthcare accessibility for people with disabilities [4]. Physical limitations may affect a person’s ability to open medication containers, remove tablets from blister packs, split tablets, measure liquid medications, use inhalers or injectable devices, apply eye drops, or manage complex medication schedules [5,6,7]. Mobility limitations may also make it difficult to visit pharmacies, pick up refills, or return for follow-up care [8]. Consequently, medication safety may be affected not only by a medication’s pharmacological properties but also by the patient’s ability to use it safely and independently, as well as by its delivery system.

People with disabilities may also face additional medication-related challenges when healthcare information and services are not adapted to their needs [9]. Standard medication labels, written instructions, dispensing counters, counseling areas, and pharmacy workflows may not adequately accommodate differences in mobility, communication, or functional abilities. Some individuals may therefore rely on family members or caregivers to collect, prepare, organize, or administer medication. Although caregiver involvement can support medication management, dependence on caregivers may also introduce medication-safety risks when responsibilities or instructions are unclear, particularly in complex medication regimens [10]. These risks may be further amplified in individuals with chronic diseases, polypharmacy, or complex medication regimens. Practical adaptations and assistive technologies may support medication self-management and independence, although evidence on their effects on clinical medication safety outcomes remains limited [11]. Pharmacists are particularly well-positioned to address medication-safety concerns related to disability. Their responsibilities extend beyond dispensing medications to include medication review, patient counseling, identification of drug-related problems, adherence support, and coordination with other healthcare professionals. For patients with disabilities, effective pharmaceutical care may require pharmacists to assess functional ability, determine whether a patient can safely use the prescribed dosage form, adapt counseling approaches, provide accessible medication information, involve caregivers when appropriate, and recommend practical strategies to support safe, independent medication use. However, pharmacists’ ability to provide such individualized care may depend on their professional training, available resources, workload, the physical pharmacy environment, and organizational support.

Pharmacists can identify medication-use difficulties, assess adherence and medication-related problems, review the suitability of dosage forms and administration devices, provide individualized counseling, and coordinate with patients, caregivers, and other healthcare professionals [12]. For people with disabilities, this role may also include assessing whether functional limitations affect the patient’s ability to obtain, understand, prepare, administer, and organize medications [13]. Such assessment may help identify practical barriers that are not apparent from a conventional medication review focused primarily on indication, dose, interactions, and adverse effects. Medication adherence is particularly relevant in this population because successful treatment depends not only on understanding and willingness to follow a regimen but also on the practical ability to manage it [14]. Disability-related functional limitations may complicate adherence by making it difficult to open packaging, manipulate dosage forms, operate administration devices, read or access medication information, organize doses, or obtain refills [15]. Adherence may also be influenced by polypharmacy, regimen complexity, caregiver dependence, communication barriers, transportation difficulties, medication costs, and the accessibility of pharmacy services [15]. These factors may interact, making apparent non-adherence partly a consequence of medication-use demands that are poorly matched to the patient’s functional capabilities.

Previous research has often focused on general healthcare accessibility, communication difficulties, or physical access to services, while comparatively less attention has been given to the relationship among functional limitations, medication-use difficulties, caregiver dependence, pharmacy environments, and pharmacists’ professional practices. Understanding these interactions is important because medication-related harm may arise not from disability itself but from a mismatch between patients’ functional needs and how medications, information, and pharmacy services are designed and delivered. In Saudi Arabia, improving accessibility and inclusion for people with disabilities is an increasingly important component of healthcare development. Nevertheless, there remains a need to understand how pharmacists encounter disability-related medication safety challenges in routine practice and whether current pharmacy services adequately meet these patients’ needs. Exploring pharmacists’ experiences can identify practical barriers, existing approaches to individualized care, and opportunities to improve professional training, accessible medication information, service design, and institutional policies.

Previous Saudi qualitative research has examined pharmacists’ perspectives on the accessibility of pharmacy services for people with disabilities, identifying barriers related to physical infrastructure, communication, professional training, assistive technologies, and service organization [16]. This study focuses on the functional demands of medication use, including medication handling, dosage-form and device use, self-administration, medication organization, caregiver-mediated use, adherence-related concerns, and continuity of access to medications. It also examines pharmacists’ views on how disability-related functional limitations affect daily medication use and raise potential medication-safety concerns. Therefore, this study aimed to explore pharmacists’ experiences and perceptions regarding medication safety and pharmaceutical care for patients with disabilities in Saudi Arabia. Specifically, the study identified disability-related medication-use challenges and perceived medication-safety concerns; described pharmacists’ approaches to individualized care; and identified training, resources, policies, and system-level strategies to strengthen disability-inclusive pharmacy practice.

## 2. Methods

### 2.1. Study Design

This study used a qualitative descriptive design to explore how disability-related functional limitations may affect safe medication use and to identify medication-related adaptations that could support safer, more independent medication management. Data were collected through a self-administered electronic survey that combined structured participant-characteristic items with open-ended questions. A qualitative descriptive approach was selected because the study required practice-based accounts of medication handling, administration, communication, self-management, medication safety risks, and adaptations, without imposing a predetermined theoretical model.

**Figure 1:**
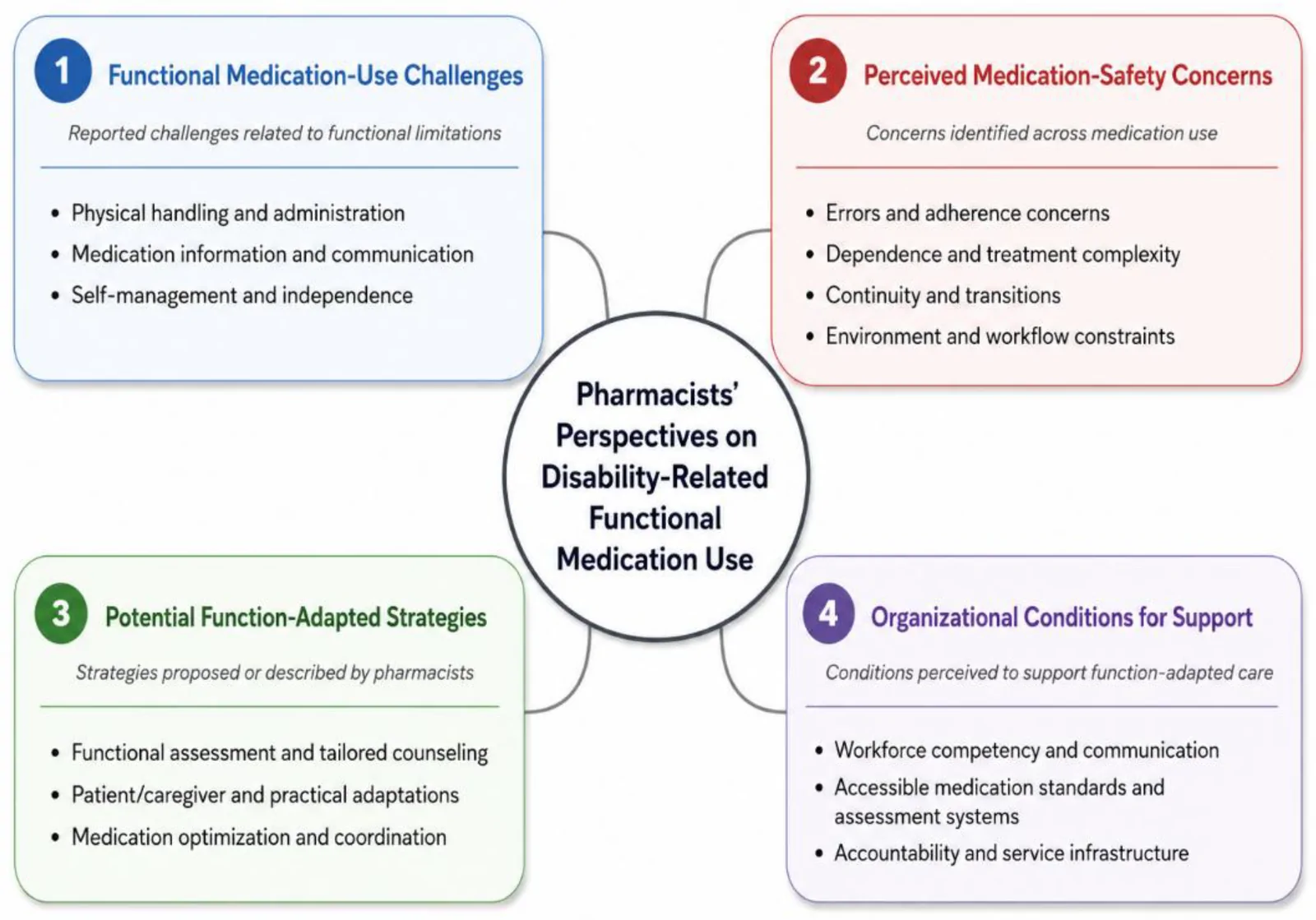
Thematic map of pharmacists’ perspectives on disability-related functional medication use.

Qualitative data were analyzed using a codebook thematic analysis approach within a qualitative descriptive orientation, informed by Braun and Clarke’s phases of thematic analysis. The thematic map presented in Figure 1 provides a visual summary of the four analytical domains identified from participants’ accounts. Reporting was guided by the Standards for Reporting Qualitative Research (SRQR). Because the study did not involve an intervention, treatment allocation, or comparison group, randomization and experimental blinding were not applicable. No a priori statistical power calculation was performed because the study was qualitative and not designed to test statistical hypotheses.

### 2.2. Study Setting and Participants

The study targeted pharmacists practicing in healthcare settings in Saudi Arabia. A non-probability sampling approach was used, and the survey invitation was distributed electronically via email, WhatsApp, and X (formerly Twitter). Because the invitation was circulated through multiple electronic channels and could be forwarded or viewed by an unknown number of individuals, the total number of pharmacists approached or who declined participation could not be determined, and a response rate could not be calculated. Participants were eligible if they were pharmacists actively practicing in a healthcare setting in Saudi Arabia, were 18 years or older, and provided electronic informed consent. Prior experience caring for people with disabilities was not required because the study also sought to capture variation in professional exposure and preparedness. Individuals who were not actively practicing pharmacists or who did not provide informed consent were excluded. Eligibility was based on self-report, and no external verification of professional credentials was undertaken. Specific employing institutions and geographic regions were not collected to preserve participant anonymity and therefore could not be reported retrospectively.

Disability-related functional limitations were operationally defined to include mobility, dexterity or upper-limb, sensory, and communication limitations that could affect medication access, understanding, handling, administration, or self-management. Accordingly, the term “people with disabilities” is used throughout the manuscript to reflect the range of functional limitations represented in the survey. A total of 18 pharmacists completed the survey and were included in the descriptive analysis. Participants represented hospital, primary healthcare, community, and clinical pharmacy settings and varied in professional experience and educational qualifications. Fifteen participants reported prior experience providing care to people with disabilities. Seventeen participants provided substantive open-text responses and were included in the qualitative analysis. One participant reported no relevant experience and provided only non-substantive open-text responses; this participant was retained in the descriptive analysis but excluded from theme development. No substantive response was excluded for differing from predominant views. The participants and dataset used in the present study were entirely independent of those included in the previously published Saudi qualitative study [16].

### 2.3. Survey Instrument and Data Collection

The electronic questionnaire included five structured participant-characteristic items and 15 open-ended questions. The research team developed the questionnaire to address the study objectives and the relevant domains identified in the literature on disability-inclusive pharmacy care and medication safety. It was organized around broad domains aligned with the study objectives, including medication-use challenges, perceived safety concerns, barriers, professional practice, and potential improvements. These domains guided data collection and provided an initial focus for the analysis. Responses were coded across the full dataset, with codes, subthemes, and themes derived from participants’ responses rather than directly from the survey questions. The questionnaire was administered in English, so no translation procedures were required. No formal pilot testing was conducted. The structured items assessed current practice setting, years of pharmacy practice, highest pharmacy qualification, prior experience caring for people with disabilities, and the most commonly encountered type of disability. The open-ended questions explored participants’ experiences providing medication-related care; medication-safety challenges; factors perceived to increase vulnerability to unsafe medication use; environmental barriers; the influence of workload, time constraints, privacy, and available resources on counseling; perceptions of current service accessibility; training, tools, and assistive services; and policies or guidelines that could support safer care. The open-ended format allowed participants to describe their experiences in their own words rather than selecting from predefined response categories.

Data were collected electronically from June to July 2026. After data collection, responses were exported to a structured dataset, checked for completeness, and anonymized. Participants were assigned identifiers P01–P18, and no directly identifying information was used during analysis. The sample size reflected the qualitative and exploratory nature of the study rather than a target for statistical representativeness. Across the 17 participants included in the qualitative analysis, the 15 open-ended questions generated approximately 5481 words of substantive qualitative text. Response length varied considerably, ranging from approximately 50 to 2443 words, with a median of approximately 133 words. Sample adequacy was assessed in relation to the focused research aim, the specificity and professional relevance of the participant group, the amount and relevance of the qualitative material generated, and the recurrence of patterns across responses. Because response depth varied substantially, longer responses were not given greater analytic weight solely because of their length. Because recruitment was not conducted iteratively until saturation, thematic saturation was not claimed. Electronic informed consent was obtained from all participants before accessing the survey. The complete questionnaire, including all open-ended questions used in the qualitative analysis, is provided as a Supplementary File.

### 2.4. Qualitative Data Analysis

The analysis had a pragmatic, experiential focus, treating responses as accounts of professional experience and perceptions rather than as objective evidence of medication-safety events. Qualitative responses were analyzed using a codebook thematic analysis approach within a qualitative descriptive orientation. The analysis was informed by Braun and Clarke’s phases of familiarization, coding, theme development, theme review, theme definition, and reporting. Participants’ responses were treated as pharmacists’ accounts and interpretations of practice rather than as direct verification of medication-safety outcomes. Although the questionnaire addressed broad domains related to medication use, safety concerns, barriers, and potential improvements, the qualitative dataset was analyzed across questions rather than on a question-by-question basis. Analysis began with repeated reading of all substantive responses to develop familiarity with the dataset. Meaningful segments of text were assigned descriptive codes without using a predefined code list. Two researchers initially coded the substantive qualitative responses independently and subsequently compared their codes, definitions, and interpretations. Differences were discussed, overlapping codes were merged, distinctions between closely related concepts were clarified, and the coding framework was progressively refined. A structured codebook was developed iteratively as the analysis moved from descriptive codes to broader patterns of meaning.

The final codebook comprised 33 codes organized into 13 subthemes and four major themes: (1) functional limitations shape perceived medication-use challenges; (2) pharmacists perceive safety concerns at multiple points in medication use; (3) proposed function-adapted strategies for medication management; and (4) organizational conditions perceived to support function-adapted care. Themes were defined according to conceptual coherence, relevance to the research question, and their capacity to represent meaningful patterns across participants’ accounts. A participant-level coding matrix was maintained to support analytic traceability and to document the relationships among participants, codes, and source responses. Participant counts were not used as thresholds for retaining themes or as indicators of thematic importance. Divergent and positive cases were actively examined during theme development. Responses that differed from prevailing patterns were retained when they challenged, refined, or added nuance to the developing interpretation. Examples of existing supportive practices, such as dedicated service arrangements and medication-delivery services, were similarly retained. Representative quotations were selected after theme development to demonstrate the relationship between the interpretations and participants’ original accounts. Quotations were identified using anonymized participant codes. Microsoft Excel (Microsoft Corporation, Redmond, WA, USA; version 2508) was used to organize the qualitative dataset, codebook, coding matrix, and supporting analytical records.

### 2.5. Quality of the Qualitative Analysis

Several procedures were used to strengthen the transparency, credibility, dependability, and confirmability of the analysis. A structured codebook and audit trail documented code definitions, changes in code boundaries, relationships among codes and subthemes, and representative source quotations. Developing interpretations were repeatedly checked against the original responses, and codes and themes were revised when the source material indicated that concepts overlapped, required clearer distinction, or did not adequately represent participants’ accounts.

Independent initial coding by two researchers was used to expose the developing analysis to multiple interpretations rather than to serve as a statistical test of coding agreement. Differences in interpretation were discussed and used to refine code definitions and thematic boundaries. Both commonly expressed and divergent perspectives were retained, and contradictory responses were examined to determine whether they challenged or modified the emerging thematic structure. Positive examples of existing practices were also considered alongside accounts of barriers and difficulties.

The researchers had backgrounds in pharmacy, pharmacology, and pharmaceutical care, which provided relevant knowledge. To reduce the influence of prior assumptions, coding was conducted across the full dataset rather than by questionnaire sections, with themes checked against participants’ responses. No formal intercoder reliability measures, like Cohen’s kappa, were used because coding was interpretive rather than quantitative. No clinical, supervisory, or other dependent relationships existed between the researchers and participants.

### 2.6. Descriptive Analysis

Structured participant characteristics were summarized with frequencies and percentages. No inferential statistical tests were conducted because these variables were used solely to characterize the qualitative study sample. All 18 respondents were included in the descriptive analysis, whereas the thematic analysis was based on the 17 participants who provided substantive qualitative data. This distinction was maintained throughout the analysis and reporting.

### 2.7. Ethical Considerations

Ethical approval was obtained from the Biomedical Research Ethics Committee at Umm Al-Qura University, Makkah, Saudi Arabia (Approval No. HAPO-02-K-012-2026-05-3464). Participation was voluntary, and responses were analyzed anonymously using participant identifiers rather than personal information. No directly identifiable personal information was included in the analytical dataset. Electronic informed consent was obtained from all participants before participation. Participants were informed about the purpose of the study, confidentiality of their responses, and their right to decline or discontinue participation without consequence.

## 3. Results

### 3.1. Participant Characteristics

A total of 18 pharmacists completed the survey and were included in the descriptive analysis. Most participants worked in hospital pharmacies (*n* = 12, 66.7%). Seven participants (38.9%) had more than 10 years of pharmacy practice experience, and nine (50.0%) held a master’s degree. Fifteen participants (83.3%) reported prior experience caring for people with disabilities. Wheelchair users were the most commonly encountered group (*n* = 10, 55.6%), followed by individuals with mobility impairments or difficulty walking (*n* = 3, 16.7%). Participant characteristics are presented in Table 1. Seventeen participants provided substantive open-ended responses and were included in the thematic analysis. One participant who reported no relevant experience and provided no substantive qualitative information was retained in the descriptive sample but did not contribute to theme development.

### 3.2. Overview of Thematic Findings

Participants’ accounts differed in their evidentiary basis. Some responses described difficulties or medication-use problems that pharmacists directly observed in practice, whereas others reflected professional judgments about potential risks or proposed strategies. The study did not independently verify medication errors, adherence outcomes, administration technique, or patient harm. Accordingly, the findings below are presented as pharmacists’ reported observations and perceptions rather than as confirmed medication-safety outcomes. The thematic analysis identified four major themes: (1) functional limitations shape perceived medication-use challenges; (2) pharmacists perceive safety concerns at multiple points in medication use; (3) proposed function-adapted strategies for medication management; and (4) organizational conditions perceived to support function-adapted care. Thirteen subthemes were identified across these four themes. The themes, key findings, and representative quotations are summarized in Table 2, while the detailed thematic framework and underlying codes are presented in Table 3. Figure 1 presents a thematic map of the four domains identified across participants’ accounts. The domains are presented as related areas of pharmacists’ perspectives and do not imply a temporal sequence, causal relationship, or demonstrated effectiveness of the proposed strategies.

### 3.3. Theme 1: Functional Limitations Shape Perceived Medication-Use Challenges

The first theme highlighted how functional ability influences everyday medication use. Participants described difficulties stemming from reduced dexterity, grip, coordination, and mobility that could interfere with opening medication containers, handling tablets, measuring liquids, or using medication devices. One participant explained: “I frequently observe adherence drops caused by the physical inability to open standard safety caps, split tablets, or accurately measure liquids. Additionally, there are often challenges with proper administration techniques for devices like inhalers due to reduced fine motor function.” (P02)

Medication information and communication were another important component of functional medication use. Participants described difficulties with inaccessible medication labels, written instructions, and medication-related communication. As P12 stated, standard counseling and prescription labels were “not fully accessible,” potentially contributing to misunderstandings about medication instructions. Participants also reported reduced independence in medication self-management. Functional limitations could affect patients’ ability to organize, prepare, administer, or manage medicines without assistance. P06 summarized this challenge by noting that some patients wished to manage their medicines independently but found this difficult because of their disability.

### 3.4. Theme 2: Pharmacists Perceive Safety Concerns at Multiple Points in Medication Use

The second theme described pharmacists’ perceptions of medication-safety concerns across multiple points in the medication-use process. Participants described missed doses, incorrect medication administration, dosing errors, and other forms of unsafe medication use. One participant reported: “The most common challenges I noticed are missed doses, confusion between medications, difficulty opening packages or handling tablets…” (P05). Dependence on caregivers and treatment complexity were also identified as potential sources of risk. Polypharmacy and complex treatment regimens increased the demands of medication management, while reliance on caregivers could create additional opportunities for miscommunication or dosing errors. P02 described “a heavy reliance on multiple, shifting caregivers,” which could increase the risk of miscommunication about dosing schedules.

Continuity of medication use was another concern. Participants described mobility-related difficulties with refills or follow-up, as well as perceived risks associated with medication changes during transitions of care. P05 noted that some patients had difficulty attending follow-up visits or obtaining refills because of transportation or mobility issues. Environmental and workflow factors were also described as potential contributors to medication-safety concerns, including physical barriers, workload, insufficient counseling time, lack of privacy, and limited resources. These factors were viewed as reducing opportunities for individualized assessment and counseling. P02 explained that heavy workloads and strict time constraints could leave insufficient time to assess whether a patient was physically able to handle a medication.

### 3.5. Theme 3: Proposed Function-Adapted Strategies for Medication Management

The third theme focused on strategies used or proposed to better align medication management with patients’ functional capabilities. Participants emphasized assessing what patients could actually do with their medicines before determining how counseling or medication support should be provided. P06 stated: “I usually focus on identifying the patient’s functional limitations before counseling.” Participants also described practical adaptations involving patients and caregivers, including caregiver education, easier packaging, assistive medication tools, and adjustments to dosage forms or medication regimens. P05 described simplifying counseling, checking whether the patient could use the dosage form correctly, and ensuring that either the patient or the caregiver understood the medication instructions. Medication review, optimization, interprofessional coordination, and continuity strategies formed the third subtheme. These strategies were viewed as ways to help ensure that medication regimens were not only clinically appropriate but also practical for the patient to use. One clinical pharmacist described reviewing medication appropriateness, identifying drug-related problems, and optimizing dosing as part of this process.

### 3.6. Theme 4: Organizational Conditions Perceived to Support Function-Adapted Care

The fourth theme reflected the view that safe medication use should not depend solely on individual pharmacists’ actions. Participants emphasized the need for workforce competency and effective communication, particularly training that addresses how disability-related functional limitations may affect medication use rather than focusing only on medication choice. As P06 stated: “Pharmacists should be trained to assess how the patient’s disability affects medication use, not only the drug choice.” Participants also proposed accessible medication standards and assessment systems, including accessible packaging and medication information, functional assessment checklists, documentation of functional needs, and standardized medication-safety procedures. P12 specifically proposed standardized, accessible packaging alternatives, such as easy-open containers and large-print labels. Accountability and service infrastructure were other important components of this theme. Participants described the need for institutional policies, accessibility standards, auditing, alternative medication-access models, and adequate resources. Existing supportive practices were also identified; for example, P11 reported that home medication delivery was available, reducing the need for some patients to attend the pharmacy in person. Overall, the findings suggest that pharmacists perceived safe medication use among people with disabilities as shaped by the interaction among functional capabilities, medication-related demands, and the systems through which medication care is delivered.

## 4. Discussion

This study provides an understanding of medication safety for people with disabilities from the perspective of practicing pharmacists in Saudi Arabia. The findings indicate that pharmacists perceived medication-use concerns when the practical requirements of treatment did not align with patients’ functional abilities. Participants also proposed several potentially useful strategies, including functional assessment, individualized counseling, adapted packaging and dosage forms, medication review, caregiver education, and stronger institutional support. A major finding was that pharmacists perceived disability-related functional limitations as potentially affecting the practical usability of medicines. Participants described difficulties opening containers, handling tablets, measuring liquids, and operating devices such as inhalers and injection devices. These findings suggest that prescribing an appropriate medicine is only one component of safe therapy. The practical suitability of treatment may also depend on whether the patient can prepare and administer it, given their functional abilities. This is particularly important for medications requiring fine motor control or repeated administration. These findings are consistent with evidence showing that difficulty opening medication packaging may contribute to medication mismanagement and poor adherence [7]. Saudi research involving people with visual impairment has similarly shown difficulties identifying medications and understanding medication information, with some patients relying on family members or caregivers for assistance [17,18]. Although visual impairment differs from mobility or dexterity limitations, these studies support the broader principle that medication systems should accommodate patients’ functional abilities. The current findings expand on previous Saudi research on pharmacy services for people with disabilities. Challenges such as physical access limitations, communication barriers, insufficient training, and a lack of assistive technologies in hospital pharmacy services have been identified [16]. Even after receiving counseling, patients may encounter medication safety issues if the packaging, dosage form, administration method, or dosing schedule proves challenging to manage independently.

Communication and medication information were also major findings. Previous studies in Saudi Arabia have reported communication difficulties between pharmacists and people with hearing impairment and have highlighted limited training in specialized communication methods [16,19]. Similar concerns were identified in an earlier Saudi qualitative study of pharmacy services for people with disabilities, in which participants emphasized communication training and assistive communication tools. However, the present findings suggest that communication should extend beyond simply delivering medication information. Pharmacists may also need to assess whether patients can follow the instructions provided. For example, understanding how to use an inhaler does not ensure that a patient with limited hand function can physically operate the device correctly. Medication-related dependence on caregivers was another important issue. Participants viewed caregiver involvement as valuable but also identified potential risks when responsibilities were unclear or when multiple caregivers were involved. Such situations may create inconsistencies in medication preparation, administration, or timing. Therefore, caregiver participation needs to be structured rather than assumed to be inherently protective. Pharmacists could clarify who is responsible for medication administration, provide counseling to both the patient and the caregiver, and verify that each person understands the treatment plan. These measures may be particularly crucial when patients are experiencing polypharmacy or intricate dosing regimens.

Mobility limitations may also affect medication safety through disrupted access to medicines and follow-up services. Participants described possible delays in prescription refills, difficulty attending pharmacies, and challenges during transitions between hospital and home. Previous regional research has mainly described these issues as accessibility barriers [16,20,21]. The present findings suggest that the consequences may extend further: difficulty reaching the pharmacy can contribute to treatment interruptions, missed doses, delayed medication reviews, and fewer opportunities for counseling. Therefore, solutions such as medication synchronization, longer refill periods when clinically appropriate, home delivery, authorized caregiver collection, and telepharmacy follow-up may have medication-safety benefits in addition to improving convenience. These approaches were proposed as potential strategies for addressing access-related medication-use concerns, although their effects on medication-safety outcomes require prospective evaluation. The findings further indicate that environmental and organizational factors can influence pharmacists’ ability to identify functional medication-use problems. High workloads, limited counseling time, inadequate privacy, and unsuitable physical spaces may reduce opportunities to evaluate whether patients can use their medicines correctly. Similar organizational barriers have been reported in other qualitative pharmacy research from Saudi Arabia. For example, pharmacists discussing prenatal medication safety also identified limitations related to training, clinical resources, communication, and institutional guidance [22]. Although the clinical populations differ, both studies suggest that individualized medication care is difficult to provide consistently without adequate organizational resources.

An important implication of the present study is the potential value of integrating functional medication-use assessment into routine pharmaceutical care. Pharmacists should assess indications, doses, interactions, and adherence, and evaluate whether the patient can physically use the prescribed treatment [15,23]. Relevant questions might include whether the patient can open the package, manipulate the dosage form, operate the administration device, access medication instructions, organize doses, and obtain refills [15,23]. When problems are identified, the pharmacist could recommend alternative dosage forms, easier-to-use packaging, assistive devices, simplified regimens, or appropriate caregiver support. In this way, medication review would include not only clinical appropriateness but also practical suitability. Importantly, function-adapted medication strategies should not be assumed universally appropriate or risk-free. Any modification to packaging, dosage form, or administration method requires product-specific clinical assessment. Easy-open containers may reduce child resistance and therefore be unsuitable in some households. Similarly, splitting tablets or changing dosage forms might be unsuitable for medicines with modified-release properties, enteric coatings, hazardous properties, or other formulation sensitivities. Repackaging can also affect the product’s stability, label accuracy, and safe identification [24,25,26]. Consequently, such adaptations should be individualized and implemented only after a pharmacist’s or prescriber’s review of the medicine, formulation, patient circumstances, and relevant safety requirements.

Participants also emphasized the importance of organizational support for function-adapted care. Participants frequently recommended professional training, accessible medication information, standardized assessment procedures, improved documentation of patients’ functional needs, and alternative medication-access services. Previous Saudi research has similarly called for disability-related education for pharmacists, service redesign, and assistive technologies [16,20]. The current study suggests that training should specifically address the relationship between functional ability and medication use. Pharmacists could benefit from practical instruction on identifying difficulties with dexterity, medication devices, dosage forms, self-administration, caregiver involvement, and medication organization.

From a policy perspective, functional medication needs could be incorporated into medication-safety procedures and electronic pharmacy records. Documenting relevant needs would enable pharmacists and other healthcare professionals to identify them during future encounters, rather than relying on patients to repeatedly report the same difficulties. Pharmacy services could also establish procedures for providing adapted packaging, accessible medication information, additional counseling, and alternative collection or delivery options when required. Such measures could help move disability-related medication support from individual pharmacist initiative toward a more consistent system of care.

Several limitations should be considered. The small, nonprobability sample limits generalizability to all pharmacists in Saudi Arabia. The questionnaire was not pilot-tested, possibly affecting the clarity of open-ended questions. Most participants practiced in hospitals, underrepresenting community and primary healthcare pharmacies. Data collection via an electronic open-ended questionnaire lacked follow-up or depth compared to interviews or focus groups. The study reflected only pharmacists’ perspectives, excluding people with disabilities and caregivers. Patients and caregivers may have different priorities and notice medication-use difficulties that pharmacists miss. Thus, findings do not establish patients’ needs or the impact of proposed adaptations on safety or independence. Future studies should include patient and caregiver views and evaluate implementation outcomes. Similar limitations are common in other qualitative pharmacy surveys.

## 5. Conclusions

This qualitative study identified pharmacists’ perceptions of functional medication-use challenges among people with disabilities, including difficulties with medication handling, administration, information, self-management, caregiver involvement, and continuity of access. Participants also proposed functional assessment, tailored counseling, medication and packaging adaptations, caregiver support, and organizational changes as potential approaches to addressing these challenges. These findings reflect pharmacists’ reported experiences and professional perceptions and should not be interpreted as evidence that the proposed strategies improve medication-safety outcomes. Rather, they provide hypotheses and practice considerations that warrant evaluation with people with disabilities, caregivers, and other healthcare professionals through prospective studies and directly measured medication-safety outcomes.

## Figures and Tables

**Table 1:** Characteristics of study participants (N = 18).

Characteristic	Category	*n* (%)
**Current pharmacy practice setting**	Hospital pharmacy	12 (66.7)
	Primary healthcare center	2 (11.1)
	Community pharmacy	2 (11.1)
	Clinical pharmacy	2 (11.1)
**Years of pharmacy practice experience**	Less than 1 year	1 (5.6)
	1–5 years	6 (33.3)
	6–10 years	4 (22.2)
	More than 10 years	7 (38.9)
**Highest pharmacy qualification**	Bachelor’s degree	2 (11.1)
	PharmD	6 (33.3)
	Master’s degree	9 (50.0)
	PhD	1 (5.6)
**Provided care to people with disabilities**	Yes	15 (83.3)
	Not sure	2 (11.1)
	No	1 (5.6)
**Type of disability most commonly encountered**	Wheelchair users	10 (55.6)
	Mobility impairment or difficulty walking	3 (16.7)
	Hearing impairment or deafness	1 (5.6)
	Speech or communication difficulty	1 (5.6)
	Visual impairment or blindness	1 (5.6)
	Had not encountered people with disabilities	2 (11.1)

**Table 2:** Major themes, subthemes, key findings, and representative quotations.

Major Theme	Subtheme	Key Finding	Representative Quotation
**Theme 1. Functional limitations shape perceived medication-use challenges**	**Physical handling and administration**	Reduced dexterity, grip, coordination, or mobility can make medication handling and administration difficult, including opening containers, manipulating tablets, measuring liquids, and using medication devices.	“I frequently observe adherence drops caused by the physical inability to open standard safety caps, split tablets, or accurately measure liquids. Additionally, there are often challenges with proper administration techniques for devices like inhalers due to reduced fine motor function.” (P02)
	**Medication information and communication**	Medication labels, written instructions, and conventional counseling may not be functionally accessible, increasing the risk of misunderstanding medication information.	“Standard counseling and written prescription labels are often not fully accessible, leading to potential misunderstandings regarding look-alike/sound-alike (LASA) medications or high-alert drug instructions.” (P12)
	**Self-management and independence**	Functional limitations may restrict patients’ ability to independently organize, prepare, administer, and manage their medicines.	“Some patients want to manage their medicines by themselves, but their disability may make this difficult.” (P06)
**Theme 2. Pharmacists perceive safety concerns at multiple points in medication use**	**Errors and adherence consequences**	Functional medication-use difficulties may translate into missed doses, incorrect administration, dosing errors, or unsafe medication use.	“The most common challenges I noticed are missed doses, confusion between medications, difficulty opening packages or handling tablets…” (P05)
	**Dependence and treatment complexity**	Polypharmacy and complex regimens increase medication-management demands, while dependence on multiple or changing caregivers can create additional opportunities for dosing and communication errors.	“A heavy reliance on multiple, shifting caregivers… significantly increases the risk of miscommunication regarding dosing schedules.” (P02)
	**Continuity and transitions**	Mobility restrictions may delay refills and follow-up, while medication changes during admission, transfer, or discharge can increase the risk of medication reconciliation and dosing errors.	“Some patients also struggle with follow-up visits or refills because of transportation or mobility problems…” (P05)
	**Environment and workflow amplifiers**	Physical environments, workload, insufficient time, lack of privacy, and limited resources may prevent adequate assessment of functional medication-use needs and individualized counseling.	“Heavy workloads and strict time constraints often rush the counseling process, leaving insufficient time to properly assess a patient’s physical capability to handle a medication.” (P02)
**Theme 3. Proposed function-adapted strategies for medication management**	**Functional assessment and tailored counseling**	Pharmacists described assessing what patients can practically do with their medicines and adapting counseling to their functional capabilities.	“I usually focus on identifying the patient’s functional limitations before counseling.” (P06)
	**Patient/caregiver and practical adaptations**	Safe medication use may require caregiver education, easier packaging, assistive medication tools, and adaptation of dosage forms or regimens.	“I usually try to make the counseling simple and practical… checking if they can use the dosage form properly, and making sure they or their caregiver understand the instructions clearly.” (P05)
	**Medication optimization and coordination**	Medication review, regimen optimization, interprofessional collaboration, and continuity strategies were viewed as mechanisms to ensure medication plans were clinically appropriate and functionally usable.	“My role includes reviewing medication regimens, assessing medication appropriateness, identifying drug-related problems, optimizing dosing…” (P14)
**Theme 4. Organizational conditions perceived to support function-adapted care**	**Workforce competency and communication**	Participants emphasized training pharmacists to recognize how functional limitations affect medication use and to adapt communication accordingly.	“Pharmacists should be trained to assess how the patient’s disability affects medication use, not only the drug choice.” (P06)
	**Accessible medication standards and assessment systems**	Participants proposed standardized, accessible packaging and information, functional assessment checklists, documentation of functional needs, and medication safety procedures.	“Policies requiring pharmacies to offer standardized, accessible packaging alternatives (e.g., easy-open containers, large-font labels)…” (P12)
	**Accountability and service infrastructure**	Institutional policies, accessibility standards, audits, alternative access models, and resource support were viewed as necessary to make functional adaptations routine and sustainable.	“There is a home delivery service for medication, eliminating the need to obtain it from a pharmacy.” (P11)

**Table 3:** Detailed thematic framework and underlying codes.

Major Theme	Subtheme	Underlying Codes
**Theme 1. Functional limitations shape perceived medication-use challenges**	**1.1 Physical handling and administration**	Medication handling and dexterity difficulty; difficulty using medication devices or dosage forms; medication packaging mismatch
	**1.2 Medication information and communication**	Inaccessible medication information; medication communication difficulty
	**1.3 Self-management and independence**	Medication organization and self-management difficulty; reduced medication-use independence
**Theme 2. Pharmacists perceive safety concerns at multiple points in medication use**	**2.1 Errors and adherence consequences**	Missed doses and reduced adherence; incorrect medication administration or dosing; medication misuse or overuse
	**2.2 Dependence and treatment complexity**	Caregiver-related medication risk; regimen complexity and polypharmacy
	**2.3 Continuity and transitions**	Mobility-related interruption of medication access; transitions-of-care medication risk
	**2.4 Environment and workflow amplifiers**	Environment as a medication-safety amplifier; workload, time, privacy, and resource constraints
**Theme 3. Proposed function-adapted strategies for medication management**	**3.1 Functional assessment and tailored counseling**	Functional medication-use assessment; tailored practical medication counseling
	**3.2 Patient/caregiver and practical adaptations**	Patient and caregiver medication education; adapted packaging and assistive medication tools; dosage-form or regimen adaptation
	**3.3 Medication optimization and coordination**	Medication review and optimization; interprofessional coordination; continuity and access adaptations
**Theme 4. Organizational conditions perceived to support function-adapted care**	**4.1 Workforce competency and communication**	Functional medication-safety competency training; communication competency training
	**4.2 Accessible medication standards and assessment systems**	Accessible medication–information and packaging standards; standardized functional assessment protocols; documentation and flagging of functional needs; standardized medication-safety and counseling procedures
	**4.3 Accountability and service infrastructure**	Institutional accessibility and accountability; system-supported alternative access models; resource and reimbursement support

## Data Availability

The data presented in this study are available on request from the corresponding author due to privacy and confidentiality considerations associated with the qualitative participant responses.

## Supplementary Materials

The following supporting information can be downloaded at: https://www.mdpi.com/article/10.3390/healthcare14182950/s1, File S1: Study questionnaire; Table S1: Detailed qualitative codebook.
