# Supplementary material for: Pharmacists’ Perspectives on Functional Medication-Use Challenges and Potential Adaptations for People with Disabilities in Saudi Arabia: A Qualitative Study
Source: Healthcare (Basel). 2026 Sep 10;14(18):2950. doi: 10.3390/healthcare14182950 (PMC13606359; doi:10.3390/healthcare14182950)
Supplement: Supplementary file 1 [file healthcare-14-02950-s001.zip › healthcare-4540877-supplementary.pdf]

SUPPLEMENTARY MATERIALS

Pharmacists’ Perspectives on Functional Medication-Use Challenges and Potential Adaptations for People with Disabilities in Saudi Arabia: A Qualitative Study

Contents

Supplementary File S1. Study questionnaire
Supplementary Table S1. Detailed qualitative codebook

Supplementary File S1. Study Questionnaire

Electronic consent and eligibility

Participation was voluntary. Before accessing the questionnaire, participants provided electronic informed consent and indicated that they were 18 years of age or older. Participants could decline participation, discontinue the survey, or skip any question.

Section A. Participant Background

1. What is your current pharmacy practice setting?

   ☐ Community pharmacy ☐ Hospital pharmacy ☐ Clinical pharmacy ☐ Primary healthcare center ☐ Academic setting

2. How many years of pharmacy practice experience do you have?

   ☐ Less than 1 year ☐ 1-5 years ☐ 6-10 years ☐ More than 10 years

3. What is your highest pharmacy qualification?

   ☐ Bachelor’s degree ☐ PharmD ☐ Master’s degree ☐ PhD ☐ Board/specialty certification

4. Have you provided care to patients with physical disabilities?

   ☐ Yes ☐ No ☐ Not sure

5. What types of physical disabilities do you most commonly encounter in your practice?

   ☐ Mobility impairment or difficulty walking ☐ Wheelchair users ☐ Visual impairment or blindness ☐ Hearing impairment or deafness ☐ Speech or communication difficulty ☐ Upper limb disability affecting medication handling ☐ Neuromuscular disorders affecting movement or coordination ☐ Amputation ☐ Stroke-related physical disability ☐ Cerebral palsy ☐ Spinal cord injury ☐ I have not encountered patients with physical disabilities in my practice.

Note: Age and sex were not collected in the administered questionnaire.

Section B. Medication Safety Experience

6. Can you describe your experience in providing medication-related care to patients with physical disabilities?
7. What medication safety challenges do you commonly observe among these patients?
8. What factors make patients with physical disabilities more vulnerable to medication errors or unsafe medication use?

Section C. Communication and Counseling

9. What communication challenges do you face when counseling patients with physical disabilities?
10. How do you adapt medication counseling for patients with visual, hearing, speech, or mobility difficulties?
11. What methods do you use to make medication instructions easier to understand?
12. Do caregivers or family members participate in counseling? Please explain when this is helpful or challenging.

Section D. Medication Use and Adherence

13. Have you encountered patients with physical disabilities who had difficulty using their medication correctly? Please describe without identifying the patient.
14. What adherence problems are most common among patients with physical disabilities?
15. How do you assess whether a patient can safely use their medication at home?

Section E. Accessibility and Practice Barriers

16. What barriers in the pharmacy environment may affect medication safety for patients with physical disabilities?
17. How do workload, time limitations, privacy, or lack of resources affect medication counseling for these patients?
18. Are current pharmacy services adequately accessible for patients with physical disabilities? Please explain.

Section F. Improvement and Recommendations

19. What training, tools, or assistive services would help pharmacists improve medication safety for these patients?
20. What policies or guidelines are needed to support safer pharmacy care for patients with physical disabilities?

Supplementary Table S1. Detailed Qualitative Codebook

Coding procedure

Open-text responses from the 15 qualitative questions were exported, checked, anonymized, and analyzed across the full dataset rather than question by question. Meaningful text segments were assigned descriptive codes without a predefined code list. Two researchers initially coded the substantive responses independently, compared codes and interpretations, merged overlapping concepts, clarified code boundaries, and iteratively refined a structured codebook. Related codes were organized into 13 subthemes and four major themes. A text segment could receive more than one code when it represented multiple concepts. Participant counts were not used as thresholds for retaining themes or as indicators of thematic importance.

Thematic hierarchy

Major theme	Subtheme	Codes
**Theme 1. Functional limitations shape perceived medication-use challenges**	1.1 Physical handling and administration	C01, C02, C03
	1.2 Medication information and communication	C04, C05
	1.3 Self-management and independence	C06, C07
**Theme 2. Pharmacists perceive safety concerns at multiple points in medication use**	2.1 Errors and adherence consequences	C08, C09, C10
	2.2 Dependence and treatment complexity	C11, C12
	2.3 Continuity and transitions	C13, C14
	2.4 Environment and workflow amplifiers	C15, C16
**Theme 3. Proposed function-adapted strategies for medication management**	3.1 Functional assessment and tailored counseling	C17, C18
	3.2 Patient/caregiver and practical adaptations	C19, C20, C21
	3.3 Medication optimization and coordination	C22, C23, C24
**Theme 4. Organizational conditions perceived to support function-adapted care**	4.1 Workforce competency and communication	C25, C26
	4.2 Accessible medication standards and assessment systems	C27, C28, C29, C30
	4.3 Accountability and service infrastructure	C31, C32, C33

Theme 1. Functional limitations shape perceived medication-use challenges

Captures pharmacists' descriptions of how disability-related functional limitations may affect everyday medication handling, administration, access to information, and independent medication management.

1.1 Physical handling and administration

Code	Code name	Operational definition	Include when the response refers to	Exclude / distinguish from
C01	Medication handling and dexterity difficulty	Difficulty physically manipulating medicines because of weakness, impaired grip, tremor, paralysis, poor coordination, or other functional limitations.	Holding tablets; splitting tablets; removing doses from packaging; measuring liquids; manipulating medicines.	General adherence problems without a physical handling component.
C02	Difficulty using medication devices or dosage forms	Difficulty correctly using a specific medication delivery device or dosage form because of functional limitations.	Inhalers; injection devices or pens; eye drops; topical medicines; patches; administration devices.	General medication handling without a specific device or administration-technique component.
C03	Medication packaging mismatch	Medication packaging is described as difficult to open, handle, or use because it does not match the patient's functional capabilities.	Child-resistant caps; blister packs; tightly sealed containers; small bottles; difficult-to-open packaging.	General physical pharmacy-access barriers; code C15 instead/additionally.

1.2 Medication information and communication

Code	Code name	Operational definition	Include when the response refers to	Exclude / distinguish from
C04	Inaccessible medication information	Medication information cannot be readily accessed or used because its format does not meet the patient's functional, sensory, or communication needs.	Small-print labels; inaccessible written instructions; difficulty identifying medicines; inaccessible warnings or directions.	General communication difficulties not specifically related to medication-information format.
C05	Medication communication difficulty	Difficulty receiving, understanding, or exchanging medication-related information because of communication or sensory limitations.	Hearing, speech, visual, or other communication barriers; misunderstanding counseling; incomplete exchange of medication information.	Problems caused primarily by regimen complexity rather than communication.

1.3 Self-management and independence

Code	Code name	Operational definition	Include when the response refers to	Exclude / distinguish from
C06	Medication organization and self-management difficulty	Difficulty independently organizing, storing, scheduling, tracking, or managing medicines in daily life.	Separating doses; organizing multiple medicines; storing medicines; managing medication schedules.	Caregiver dependence without evidence of a self-management difficulty.
C07	Reduced medication-use independence	Functional limitations reduce the patient's ability to independently prepare, administer, obtain, or manage medication.	Need for assistance; inability to self-administer; reduced independence in medication management.	Caregiver-related errors or miscommunication themselves; code C11 instead/additionally.

Theme 2. Pharmacists perceive safety concerns at multiple points in medication use

Captures pharmacists' reported observations and professional perceptions of potential medication-safety concerns associated with adherence, administration, caregiver involvement, treatment complexity, continuity, and service conditions. These codes do not represent independently verified medication-safety outcomes.

2.1 Errors and adherence consequences

Code	Code name	Operational definition	Include when the response refers to	Exclude / distinguish from
C08	Missed doses and reduced adherence	Reports or perceptions of missed, delayed, or omitted doses or reduced adherence associated with functional, access, organizational, or regimen-related challenges.	Missed doses; delayed doses; non-adherence; difficulty following the intended regimen.	Intentional misuse or overuse without an adherence context; code C10.
C09	Incorrect medication administration or dosing	Reports or perceptions of incorrect administration, incorrect dosing, or increased opportunity for an administration or dosing error.	Wrong dose; incorrect administration technique; incorrect use of medication; dosing errors.	Difficulty using a device without an identified or perceived administration error; code C02 instead/additionally.
C10	Medication misuse or overuse	Reports or perceptions that medication is used excessively, inappropriately, or differently from the intended regimen.	Misuse; overuse; inappropriate medication use.	Missed doses or unintentional non-adherence; code C08.

2.2 Dependence and treatment complexity

Code	Code name	Operational definition	Include when the response refers to	Exclude / distinguish from
C11	Caregiver-related medication risk	Reliance on caregivers is described as a potential source of medication-related problems when roles, instructions, or responsibilities are inconsistent.	Multiple or changing caregivers; insufficient caregiver education; unclear dosing responsibilities; caregiver miscommunication.	Caregiver support described only as beneficial; code C19.
C12	Regimen complexity and polypharmacy	Multiple medicines, chronic conditions, frequent dosing, changing regimens, or complicated instructions are described as increasing medication-management demands or perceived risk.	Polypharmacy; several daily doses; dose changes; complex instructions; drug-drug interaction concerns.	Difficulty manipulating an individual medicine; use C01-C03 as appropriate.

2.3 Continuity and transitions

Code	Code name	Operational definition	Include when the response refers to	Exclude / distinguish from

Code	Code name	Operational definition	Include when the response refers to	Exclude / distinguish from
**C13**	Mobility-related interruption of medication access	Mobility limitations are described as interfering with obtaining medicines, refills, or follow-up and therefore with continuity of medication use.	Difficulty reaching the pharmacy; delayed refills; missed collection; transportation problems; difficulty returning for follow-up.	Physical layout barriers that do not affect continuity; code C15.
**C14**	Transitions-of-care medication risk	Medication changes during admission, transfer, or discharge are described as potential sources of discrepancies, misunderstanding, or dosing problems.	Medication reconciliation; duplication; omission; changed medicines; discharge-related confusion.	Routine medication review outside transitions of care.

2.4 Environment and workflow amplifiers

Code	Code name	Operational definition	Include when the response refers to	Exclude / distinguish from
**C15**	Environment as a medication-safety amplifier	Physical pharmacy conditions are described as limiting medication receipt, communication, counseling, or assessment of functional medication-use needs.	High counters; narrow spaces; inaccessible windows; crowded environments; inadequate seating.	Workflow or staffing pressures without a primarily physical-environment component; code C16.
**C16**	Workflow, time, privacy, and resource constraints	Service pressures are described as limiting the pharmacist's ability to assess functional needs, counsel appropriately, demonstrate medication use, or verify understanding.	Workload; rushed counseling; insufficient time; lack of privacy; inadequate staffing; lack of materials or assistive resources.	Physical infrastructure barriers not primarily related to workflow; code C15.

Theme 3. Proposed function-adapted strategies for medication management

Captures strategies described or proposed by pharmacists to better align medication management with patients' functional capabilities. These strategies are practice considerations and are not presented as proven interventions.

3.1 Functional assessment and tailored counseling

Code	Code name	Operational definition	Include when the response refers to	Exclude / distinguish from
**C17**	Functional medication-use assessment	Assessment of what the patient can physically and practically do with medicines before or during counseling, medication review, or care planning.	Assessing dexterity, grip, mobility, ability to open containers, administer medicines, access instructions, organize doses, or self-manage.	General medication review without assessment of functional medication use.
**C18**	Tailored practical medication counseling	Counseling is adapted to the patient's functional, communication, or medication-use needs.	Simplified explanations; practical instructions; demonstrations; accessible written information; checking ability to use a dosage form.	Routine standard counseling without adaptation.

3.2 Patient/caregiver and practical adaptations

Code	Code name	Operational definition	Include when the response refers to	Exclude / distinguish from
**C19**	Patient and caregiver medication education	The patient and/or caregiver is deliberately educated or involved to support appropriate medication administration and management.	Counseling caregivers; defining medication roles; confirming patient/caregiver understanding.	Caregiver dependence described primarily as a potential risk; code C11.
**C20**	Adapted packaging and assistive medication tools	Medication-related aids or packaging adaptations are used or proposed to support medication usability or independence.	Easy-open containers; pill organizers; multi-dose packs; tablet splitters; inhaler spacers; injection aids; eye-drop guides; reminder devices.	Institutional or policy requirements for accessible packaging; code C27 instead/additionally.
**C21**	Dosage-form or regimen adaptation	A dosage form, device, administration method, or regimen is modified or proposed to better match the patient's functional capabilities.	Alternative dosage forms; simplified regimens; changing an unusable device; adapting administration method.	General clinical dose optimization unrelated to function; code C22.

3.3 Medication optimization and coordination

Code	Code name	Operational definition	Include when the response refers to	Exclude / distinguish from
**C22**	Medication review and optimization	Medication review or optimization is described as part of addressing medication-related problems, treatment complexity, or appropriateness.	Medication reconciliation; appropriateness review; dosing; adverse-event monitoring; drug-related problems; interaction review.	Functional assessment alone; code C17.
**C23**	Interprofessional coordination	Pharmacists coordinate or propose coordination with other healthcare professionals to address functional medication-use needs.	Physicians; nurses; occupational therapists; physiotherapists; multidisciplinary teams; referrals.	Caregiver-only involvement without healthcare-professional coordination.
**C24**	Continuity and access adaptations	Medication-access or follow-up arrangements are used or proposed to address mobility or continuity limitations.	Medication synchronization; longer refills when appropriate; home delivery; caregiver pickup; refill reminders; telepharmacy; post-discharge follow-up.	General physical pharmacy accessibility without a continuity intervention; code C15.

Theme 4. Organizational conditions perceived to support function-adapted care

Captures organizational and system-level conditions that pharmacists perceived as supporting more consistent function-adapted medication care, including workforce competencies, accessible standards, documentation, accountability, alternative access models, and resources.

4.1 Workforce competency and communication

Code	Code name	Operational definition	Include when the response refers to	Exclude / distinguish from

Code	Code name	Operational definition	Include when the response refers to	Exclude / distinguish from
**C25**	Functional medication-safety competency training	Training pharmacists to recognize and address how disability-related functional limitations may affect medication use and medication-related care.	Assessing dexterity or mobility; administration capability; disability-sensitive medication counseling; functional medication-use assessment.	Generic professional development unrelated to disability or medication use.
**C26**	Communication competency training	Training specifically aimed at communicating with patients who have disability-related communication or sensory needs.	Sign language; accessible communication techniques; disability-sensitive communication skills.	Training focused primarily on functional medication-use assessment; code C25.

4.2 Accessible medication standards and assessment systems

Code	Code name	Operational definition	Include when the response refers to	Exclude / distinguish from
**C27**	Accessible medication-information and packaging standards	Institutional or policy requirements to provide medication information or packaging in formats intended to be more usable for people with functional or sensory limitations.	Large-print labels; easy-open packaging; accessible instructions; tactile, pictorial, or audio information; standardized accessible packaging.	Individual provision of an aid without a policy or system dimension; code C20.
**C28**	Standardized functional assessment protocols	Formal protocols or checklists requiring functional medication-use needs to be assessed systematically.	Functional-use checklists; disability assessment during counseling or discharge; standardized medication-use assessment.	Informal individual functional assessment; code C17.
**C29**	Documentation and flagging of functional needs	Recording relevant functional medication-use needs so they can be recognized across encounters and by different healthcare professionals.	Electronic record flags; documentation of functional or accessibility needs; medication-use limitations in health or pharmacy records.	Verbal assessment without documentation.
**C30**	Standardized medication-safety and counseling procedures	Formal procedures intended to promote consistent medication-related care for people with functional limitations.	Medication-safety guidelines; counseling protocols; discharge procedures; caregiver-involvement guidelines.	Individual tailored counseling without a formal procedure; code C18.

4.3 Accountability and service infrastructure

Code	Code name	Operational definition	Include when the response refers to	Exclude / distinguish from
**C31**	Institutional accessibility and accountability	Formal organizational requirements or monitoring mechanisms related to accessible medication care and service delivery.	Accessibility standards; audits; accreditation requirements; accessible counseling areas; organizational accountability.	Description of an existing barrier without an organizational response; code C15.
**C32**	System-supported alternative access models	Institutionally supported service models that reduce mobility-related barriers to medication access or pharmacy services.	Formal home-delivery programs; telepharmacy; priority pathways; dedicated dispensing windows; outreach services.	An individual suggestion for refill assistance without system-level implementation; code C24.
**C33**	Resource and reimbursement support	Organizational or policy support for staffing, funding, reimbursement, or resources needed to provide individualized or function-adapted medication care.	Additional staffing; resources for customized packaging; reimbursement for extended counseling or support services.	General lack of time or resources described as a current barrier; code C16.
